# A quantitative assessment of the level of knowledge, attitude and practices of farmworkers regarding schistosomiasis in a rural community in South Africa

**DOI:** 10.4102/phcfm.v12i1.2098

**Published:** 2020-06-08

**Authors:** Fulufhelo Nenzhelele, Felix C. Anyanwu, Mamabolo Ramoteme, Jabu Mabunda, Akinsola Henry, Kyei Kwabena

**Affiliations:** 1 Department of Public Health, School of Health Sciences, University of Venda, Thohoyandou, South Africa; 2 Applied Research for Community Development (ARCD), Limpopo, South Africa; 3 Elliot Provincial Hospital, Eastern Cape, South Africa; 4 Wits School of Public Health NGO Support, University of the Witwatersrand, Johannesburg, South Africa; 5 Department of Nutrition, University of Venda, Thohoyandou, South Africa; 6 Department of Statistics, University of Venda, Thohoyandou, South Africa

**Keywords:** assessment, knowledge, attitude, practices, farmworkers, schistosomiasis

## Abstract

**Background:**

Schistosomiasis is associated with agriculture and water development schemes, and farmworkers are particularly vulnerable to this disease because of their regular contact with water.

**Aim:**

To determine the level of knowledge, attitude and practices (KAP) of farmworkers regarding schistosomiasis.

**Setting:**

This study was conducted in Vuvha, a rural community under Makhado municipality, Vhembe district, Limpopo Province, South Africa.

**Methods:**

A quantitative, cross-sectional design was used. A self-administered questionnaire was used for data collection, and data were analysed using descriptive and inferential statistical techniques.

**Results:**

The majority were knowledgeable about the cause of schistosomiasis (84.3%), knew the mode of transmission of the disease (90.2%). However, about half of the participants knew the symptoms of schistosomiasis. Sixty-eight (33.4%) believed that schistosomiasis was not a problem in their community. The majority (77.9%) agreed that it was abnormal to pass blood in urine, while 85.8% agreed that medical consultation was the right thing to do when symptoms are observed. Fifty-five participants (27.0%) reported ever passing bloody urine. Among those who passed bloody urine, 43 (78.2%) consulted a doctor. Fifty-two (26.0%) participants reported ever being treated for schistosomiasis.

**Conclusion:**

The level of knowledge about the cause of schistosomiasis is high among the participants; similarly, there are positive attitudes and good practices shown in this study, but there are some gaps that need to be addressed. Efforts should be made to continue to educate farmworkers because they are at an increased risk for contracting schistosomiasis.

## Introduction

Schistosomiasis, commonly known as bilharzia, is caused by an intravascular trematode infection that is associated with agriculture and water schemes. This disease is most prevalent in sub-Saharan Africa, where it is one of the most common parasitic infections.^1^ The spread of schistosomiasis within communities whose health systems face difficulties in providing basic healthcare exposes such communities to poverty, increased morbidity and mortality,^2^ thereby reducing the workforce of the affected communities.^1^ Various studies have associated high prevalence of schistosomiasis with agricultural practices, especially among local farming communities.^3,4^ The impact of this is a resulting loss of income, poverty and lowered standard of living among farmworkers.^5,6^ Over the years, research has shown that farmworkers exhibit low knowledge on how schistosomiasis is transmitted and the appropriate measures to control the disease.^7,8,9,10^ Even among health care professional, the level of knowledge regarding schistosomiasis has been shown to be marginal.^11,12^ The low level of knowledge may have been influenced by some erroneous beliefs about schistosomiasis, and this may have informed the general perception regarding the severity of and the susceptibility to the disease. Hence, schistosomiasis is not perceived as a major health problem and therefore the danger posed by this disease is ignored.^8,13,14,15^ Even in communities where the level of knowledge is high, the majority of people do not take preventive measures to control the disease,^14^ and those who get infected may not avail treatment.^14,16,17^

The history of schistosomiasis in South Africa dates back to the 1900s, when the disease was shown to be endemic in the areas now known as the Eastern Cape, Kwazulu-Natal and Limpopo. The prevalence of the disease ranged between 42% and 81% from 1962 to 1977, stretching from the Venda homeland to the Komati river basin near the Mozambique border.^18^ Studies conducted in the first decade of the 21st century suggest that schistosomiasis is still endemic in South Africa, and this can be seen in the rise of the prevalence of the disease in the historically endemic areas in Mpumalanga, Kwazulu-Natal, Limpopo and the North-West Provinces.^19,20,21,22^ Most of the new infections occurred within the rural communities; this is because the disease is characteristically associated with agricultural activities, absence of piped water and lack of good sanitary facilities, which are often the hallmarks of rural communities.^23,24^

Farmworkers in these rural communities are at an increased risk of contracting schistosomiasis, but there appears to be no study on the level of knowledge, attitude and practices (KAP) regarding schistosomiasis in these rural communities in South Africa.

## Aim and objectives

The aim of this study was to determine the KAP of farmworkers regarding schistosomiasis, and this is necessary because farmworkers have an increased risk for schistosomiasis infection because of their regular contact with water. Therefore, having an insight into the level of KAP of farmworkers towards schistosomiasis may inform policy towards elimination of this disease in South Africa. The specific objectives were as follows:

1. to determine the level of knowledge of farmworkers regarding schistosomiasis
2. to determine the attitude of farmworkers regarding schistosomiasis
3. to assess the practices of farmworkers regarding schistosomiasis.

## Methodology

### Study design

This study used a quantitative, cross-sectional design, which is often used to determine the current status of population characteristics. In this study, it was used to determine the KAP regarding schistosomiasis in Vuvha community.

### Study setting

This study was conducted in Vuvha, a community in Makhado municipality, under Vhembe district, Limpopo Province, South Africa. This community has all the features of a rural setting, including low levels of literacy, poor infrastructure and perennial scarcity of water for both domestic and agricultural use. The main source of water in the community is the Mudzinga river and the health service is centered around the Vuvha clinic. Furthermore, the community is situated in one of the schistosomiasis-endemic areas in South Africa, and reports from the local clinic suggest that the disease is still endemic in the community.

### Population and sampling

This study was conducted among farmworkers within the community and because there were only 250 (140 males and 110 females) farmworkers in the employment of the three commercial farms in the community, a total population sampling was used.

### Data collection method and instrument

There are three commercial farms in the Vuvha community, namely, Entabeni, Thimbadola and Magovhoni farms. Data were collected from all the farms with the help of research assistants who helped in distributing and collecting questionnaires. Self-administered questionnaires were used for data collection, and the questionnaires were developed in English and Tshivenda languages to accommodate both the educated and the uneducated. Furthermore, the questionnaire was divided into four sections: the demographic profile of the participants, the level of Knowledge, Attitude and Practices of farmworkers regarding schistosomiasis. The validity of the instrument was ensured by extensive literature review, and the findings from this review were used to construct the questionnaire. The reliability of the instrument was determined by the test–retest method where the same questionnaire was administered to the same set of participants on day 1 and 3 days later, and a correlation coefficient of 0.82 was established.

### Data analysis

In order to determine the level of knowledge, participants responded to a set of questions designed to test their knowledge on schistosomiasis. Their responses were scored as either correct or incorrect. A score of 2 out of 5 and below was considered low knowledge, and a score of 3 and above was considered high knowledge.

Thirty-nine farmworkers declined to participate in this study, while seven questionnaires were not analysed because they were not properly completed. A total of 204 questionnaires were available for analysis, and the Statistical Package for Social Sciences (SPSS) version 20 was used to analyse the data. Frequency tables were used to present the demographic profile and the responses on KAP, while generalised linear model and multinomial logistic regression were used to measure the relationship between dependent variables and covariates. Statistical significance was set at *p* < 0.05.

### Ethical consideration

Permission to conduct the research was obtained from the Research Ethics Committee of the University of Venda (SHS/13/PH/06/0625). The procedure for this study and its aim and objectives were explained to the participants and only those who gave their consent were allowed to participate in this study.

## Results

This study involved 204 participants aged between 20 and 60 years. The mean age was 33.4 (s.d. = 9.6) years. As shown in Table 1, there were more males (55.4%) than females (44.6%), and most (64.9%) of the participants were within the 20–39 years age group, and only 38.2% of the participants were married at the time of this study. About one in five of the participants had primary school education or no formal education at all; these participants were categorised as illiterates, while the remaining participants were categorised as literates because they could read and write in English or Tshivenda. This categorisation took into account the low literacy rates in the general population.

**TABLE 1:** Demographic characteristics of the participants (*n* = 204).

Variables	Frequency	Percentage
**Gender**		
Male	113	55.4
Female	91	44.6
**Age (years)**		
20–29	69	33.3
30–39	65	31.9
40–49	50	24.5
> 50	21	10.3
**Marital status**		
Single	75	36.8
Married	78	38.2
Cohabiting	42	20.6
Widowed	9	4.4
**Education**		
Grade 0–7	41	20.1
Grade 8–10	50	25.5
Grade 11–12	94	45.1
Tertiary	19	9.3

Table 2 shows that less than half of the participants had high knowledge about the common symptoms of schistosomiasis, but the majority (84.3%) had high knowledge about the cause of the disease as well as the mode of transmission (90.2%). The majority (72.5%) also knew the geographical distribution of the disease. The knowledge on ‘who can be affected’ by the disease was also high among the participants – 80.4% knew that the disease could affect children as well as adults, and only 13.2% reported that it is a disease that affects only black people.

**TABLE 2:** Level of knowledge about schistosomiasis (*n* = 204).

Variable	Low knowledge		High knowledge	
	*n*	%	*n*	%
Common symptoms of schistosomiasis	115	56.4	89	43.6
Cause of schistosomiasis	32	15.7	172	84.3
Schistosomiasis has no cure	31	15.2	173	84.8
It is a black man’s disease	27	13.2	177	86.8
It is transmitted through contaminated water	20	9.8	184	90.2
It is common in poor riverside communities	56	27.5	148	72.5
It affects children as well as adults	40	19.6	164	80.4
It is a spiritual problem	40	19.6	164	80.4

As shown in Table 3, further analysis of key variables using generalised linear model showed that participants had no significant difference in their level of knowledge across age and gender regarding the ‘common symptoms of schistosomiasis’; however, there was significant difference in knowledge when participants were assessed along marital status, and a marginal difference along level of education but not significant at 5% margin. Considering those who had high knowledge of the common symptoms of the disease, participants who were cohabiting (*p* = 0.040) or married (*p* = 0.015) were significantly less likely to report high knowledge compared to those who were widowed, while those who had less than tertiary education were less likely to report high knowledge, especially those who attained grade 7 and below. Concerning knowledge on the cause of the disease, there was no significant relationship across age, level of education, marital status and gender, but females compared with males were more likely to report high knowledge regarding the cure for the disease (*p* = 0.031). On whether the disease affects children as well as adults, cohabiting (*p* = 0.022) and married (*p* = 0.046) participants were more likely to have high knowledge when compared to those who are widowed.

**TABLE 3:** Generalised linear model analysis of level of knowledge.

Variables	Common symptoms of schistosomiasis		Causes of schistosomiasis		Schistosomiasis has no cure		It affects children as well as adults	
	*B*	Sig	*B*	Sig	*B*	Sig	*B*	Sig
**Intercept**	2.453	0.012	2.299	0.087	21.660	0.999	21.097	0.999
**Education**								
0–7	−1.126	0.057	−0.499	0.572	−20.758	0.999	−21.643	0.999
11–12	−0.904	0.085	−0.610	0.447	−21.161	0.999	−20.994	0.999
8–10	−0.681	0.225	−0.533	0.531	−20.834	0.999	−21.250	0.999
Tertiary	Reference category for education							
**Gender**								
female	−0.381	0.205	0.631	0.131	0.941	0.031†	0.156	0.676
Male	Reference category for gender							
**Marital status**								
Cohabiting	−1.812	0.040†	−659	0.568	2.022	0.073	1.934	0.022†
Married	−2.091	0.015†	−303	0.789	0.198	0.824	1.500	0.046†
Single	−1.533	0.072	−208	0.855	0.559	0.540	1.165	0.120
Widowed	Reference category for marital status							

sig, significant; *B*, coefficient.

† , Reference category for the dependent variable is ‘low knowledge’.

As shown in Table 4, 68 (33.4%) participants disagreed that schistosomiasis is a problem of concern and 52 (25.5%) agreed, while 84 (41.2%) did not know whether it is a problem of concern or not. Regarding whether it is normal to pass blood in the stool, 140 (68.6%) disagreed while 7 (3.4%) agreed. On whether someone could get infected when she or he gets in contact with infected water source, 27 (13.2%) disagreed while 142 (69.6%) agreed. On whether there is nothing wrong with being infected with schistosomiasis, 137 (67.2%) disagreed while 15 (7.4%) agreed. With regard to the question on whether children should not go to riverside for bathing and washing purposes, 47 (23.1%) disagreed, 49 (24.0%) agreed and the remaining participants responded, ‘I do not know’.

**TABLE 4:** Attitude of farmworkers towards schistosomiasis (*n* = 204).

Concerning schistosomiasis	D		DK		A	
	*n*	%	*n*	%	*n*	%
It is not a serious problem	68	33.4	84	41.2	52	25.5
Passing blood in the urine is normal	159	77.9	33	16.2	12	5.9
Worry when there is blood in the urine	8	4.0	28	13.7	168	82.3
It is okay to pass blood in the stool	140	68.6	57	27.9	7	3.5
It is okay to bathe and wash in the stream	57	27.9	83	40.7	64	31.4
Seeking medical help is the thing to do	11	5.4	18	8.8	175	85.8
It is better to take herbal preparations	99	48.6	87	42.6	18	8.8
Blood in urine is a sign of maturity	142	69.6	49	24.0	13	6.4
Painful urination could be a sign	21	10.3	42	20.6	141	69.1
Nothing wrong with being infected	137	67.2	52	25.5	15	7.3
Children should not bathe in the river	47	23.0	118	57.8	39	19.2
No problem urinating in the stream	175	85.8	22	10.8	7	3.5

D, disagree; DK, I do not know; A, agree.

As shown in Table 5, using multinomial logistic regression, we further analysed key variables to explore possible relationships between attitude and socio-demographic factors. Participants’ attitude on whether it is a good thing to bathe and wash clothes in the stream found a significant relationship with gender and level of education. In comparison with the reference value (‘I do not know’), among those who disagreed with this statement, females were significantly (*p* = 0.045) less likely to disagree, while those with less than grade 8 education were also less likely to disagree in comparison to those with tertiary education. On ‘whether to seek medical help when infected’ found significant difference with marital status – among those who disagreed, it was shown that those cohabiting (*p* = 0.000) or married (*p* = 0.000) were more likely to disagree. Similarly, those who were cohabiting (*p* = 0.000) and those who were married (*p* = 0.000) were more likely to agree that it is better to seek help from a traditional healer when infected.

**TABLE 5:** Multinomial logistic regression analysis of the attitude of participants.

Variables	Okay to bathe and wash in the river				Seeking medical help is the right thing to do once we see blood in our urine				It is better to take herbal preparations at home				Passing blood in the urine is a sign of maturity			
	Agree		Disagree		Agree		Disagree		Agree		Disagree		Agree		Disagree	
	*B*	sig	*B*	sig	*B*	sig	*B*	sig	*B*	sig	*B*	sig	*B*	sig	*B*	sig
**Intercept**	0.901	0.525	1.070	0.458	20.506	0.996	−15.580	0.998	−17.651	0.000	−0.334	0.782	−19.963	0.000	2.816	0.039†
**Age**	−0.009	0.655	−0.002	0.921	−0.054	0.063	−0.061	0.198	0.018	0.600	0.037	0.048	0.047	0.194	−0.044	0.036†
**Gender**																
female	−0.314	0.372	−753	0.045†	0.657	0.229	−296	0.740	−1.088	0.063	−0.456	0.140	−0.870	0.220	−0.511	0.145
Male	Reference category for gender															
**Marital status**																
Cohabiting	0.281	0.775	0.491	0.612	0.673	0.507	16.27	0.000†	15.16	0.000†	0.403	0.606	17.92	0.000†	1.201	0.19
Married	−214	0.819	0.050	0.957	0.500	0.595	17.99	0.000†	17.08	0.000†	0.320	0.668	17.55	0.000†	0.870	0.25
Single	0.201	0.834	−363	0.709	0.790	0.442	17.022	−	16.899	−	0.632	0.415	18.175	.	0.871	0.29
Widowed	Reference category for marital status															
**Education**																
0–7	−1.90	0.017†	−1.87	0.016†	−16.974	0.996	0.205	1.000	−2.329	0.092	−1.53	0.026†	−1.886	0.261	−0.606	0.483
11–12	−0.497	0.464	−0.767	0.260	−17.059	0.996	0.795	1.000	−0.481	0.616	−0.925	0.137	−0.149	0.908	−1.010	0.205
8–10	−5.51	0.478	−1.23	0.094	−17.412	0.996	−17.35	0.998	−0.943	0.360	−1.34	0.041†	−0.942	0.520	−0.831	0.318
Tertiary	Reference category for Education															

sig, significant; *B*, coefficient.

† , Reference category for the dependent variable is ‘I do not know’.

Regarding whether passing blood in the urine was a sign of maturity, those who were married (*p* = 0.000) or cohabiting (*p* = 0.000) were more likely to agree, while among those who disagreed, age as a covariate showed a significantly negative relationship (*p* = 0.036). However, when age was isolated and analysed as a factor, there was no significant difference found across across age group.

As shown in Table 6, most of the water used on the farm came from the dam (52.9%) and the river (42.6%). Among the participants, 99 (48.5%) claimed that they were always in contact with water, 94 (46.1%) said they sometimes came in contact with water while at work and 11 (5.4%) said they had no contact at all with water. Fifty-five participants (27.0%) reported ever-passing blood in their urine; among these, 43 (78.2%) consulted a doctor, 6 (10.9%) consulted a traditional healer and 6 (10.9%) did nothing. Fifty-two (26.0%) participants reported having been treated for schistosomiasis; and among these, 32 (61.5%) have been treated once, 16 (30.8%) have been treated twice and 4 (7.7%) have been treated more than twice.

**TABLE 6:** The practices of farmworkers regarding schistosomiasis.

Variable	*n*	%
**How often do you work on the farm?**		
Daily	177	86.8
Thrice a week	19	9.3
Others	8	3.9
**What is the source of water for your farm?**		
Dam	108	52.9
River	87	42.6
Tap	9	4.4
**How often do you come in contact with water while working on the farm?**		
Always	99	48.5
Sometimes	94	46.1
No contact	11	5.4
**While on the farm, where do you urinate?**		
In the nearby stream	25	12.3
On the farm	60	29.4
Toilet	119	58.3
**Have you ever passed blood in your urine?**		
Yes	55	27.0
No	149	73.0
**If yes, what action did you take?**		
Consult a doctor	43	78.2
Consult a traditional healer	6	10.9
Did nothing	6	10.9
**Do you wash or bathe in the river?**		
Yes	82	40.2
No	122	59.8
**Have you ever been treated for schistosomiasis in the past?**		
Yes	53	26.0
No	151	74.0
**If yes, how many times?**		
Once	32	61.5
Twice	16	30.8
More than twice	4	7.7

Table 7 shows a multinomial regression analysis of data on the practices of participants, and our analysis reveals significant relationship with marital status and the site of urination on the farm. It was shown that married (*p* = 0.038) and single (*p* = 0.031) participants were less likely to urinate in the stream near the farm, while females (*p* = 0.004) were generally less likely to urinate on the farm. Regarding passing blood in the urine, participants who were married (*p* = 0.043) and those who were single (*p* = 0.046) were more likely to report that they have not passed blood in their urine. Among those who passed blood in their urine, married (*p* = 0.050) participants were less likely to consult with a doctor, while our finding also showed that married (*p* = 0.000) and cohabiting (*p* = 0.000) participants were more likely to consult with a traditional healer. Age, gender and level of education were not significantly associated with the practices of the participants in this study.

**TABLE 7:** Multinomial logistic regression analysis of the practices of participants.

Variables	While on the farm, where do you urinate? (RC: Toilet)				Ever passed blood in urine? (RC: Yes)		What action did you take? (RC: Did nothing)				Treated for schistosomiasis? (RC: Yes)	
	Stream		On the farm		No		A doctor		A healer		No	
	*B*	sig	*B*	sig	*B*	sig	*B*	sig	*B*	sig	*B*	sig
**Intercept**	−0.749	0.685	−1.047	0.488	−0.807	0.522	−0.145	0.919	−20.272	0.000	0.277	0.829
**Age**	0.012	0.665	0.012	0.573	0.016	0.444	−0.009	0.688	0.022	0.687	0.007	0.728
**Gender**												
female	−0.738	0.134	−1.006	0.004?	0.522	0.122	−0.658	0.080	−0.104	0.913	0.647	0.059
Male	Reference category for gende											
**Marital status**												
cohabiting	−1.346	0.189	−0.680	0.540	1.048	0.182	−1.209	0.154	17.811	0.000?	0.603	0.444
Married	−2.102	0.038?	−0.221	0.836	1.526	0.043?	−1.584	0.050?	16.790	0.000?	1.191	0.117
Single	−2.294	0.031?	−0.626	0.566	1.581	0.046?	−1.264	0.132	16.067	.	0.978	0.216
Widowed	Reference category for marital status											
**Education**												
0–7	1.852	0.116	1.391	0.077	−0.451	0.518	0.932	0.285	−0.844	0.599	−0.704	0.352
11–12	0.663	0.551	0.685	0.332	−0.131	0.832	0.514	0.524	−0.235	0.842	−0.630	0.354
8–10	1.178	0.310	1.374	0.062	−0.638	0.326	1.277	0.122	−17.290	0.996	−0.888	0.213
Tertiary	Reference category for education											

RC, Reference category; Sig, significant; *B*, coefficient.

## Discussion

Studies on the knowledge of schistosomiasis have been diverse in its findings; some authors have reported low knowledge,^25,26,27^ while others found high knowledge,^28^ but strikingly, most of the studies that found low knowledge were conducted in communities where the health systems face difficulties in providing basic care at the primary health care level. This study however found high knowledge on the cause and mode of transmission of the disease, and the level of knowledge found in this study may be related to the level of awareness among the population and also the high level of literacy among the participants in comparison to the general population. However, this study also revealed that about half of the participants had low knowledge of the associated symptoms of the disease, and this was similar to the findings of Salehe et al.^28^ This may have implications for treatment and care because if the individuals do not recognise the symptoms of the disease, they may not likely seek help. Our study did not find significant age or gender difference in the level of knowledge regarding schistosomiasis except for knowledge regarding the cure of the disease where females reported higher knowledge. This finding may be attributed to the high level of education among the participants in this study. Conversely, Onyeneho et al.^14^ found no significant gender difference in knowledge among their participants, but it was reported elsewhere that males are more knowledgeable than females regarding the disease.^27^ However, our study found a significant difference along the lines of marital status, and interestingly, the reason for this relationship is not apparent. We found that being married or cohabiting was associated with low knowledge regarding symptoms of the disease but high knowledge regarding the cure of the disease, while having tertiary education seemed to increase the level of knowledge. The latter seems to appeal to common sense because it is expected that when people attain higher level of education, they get more exposure and therefore more critical expression of cognitive functions.

The attitude towards the prevention and control of schistosomiasis among the participants in this study was found to be good, especially concerning passing blood in the urine and medical consultation, but some participants did not have a problem with bathing and washing in the stream. Washing and bathing in the stream demonstrates a negative attitude towards preventing schistosomiasis because this increases water contact and thereby the risk of infection. However, the attitude of the participants in our study was better than that reported by Tang et al.,^29^ where the overall attitude towards schistosomiasis control was poor. Similarly, Onyeneho et al.^14^ also found poor attitude regarding schistosomiasis; in their study, the majority of the participants did not seek medical treatment because of the belief that there was no effective cure for the disease and because schistosomiasis infection was considered a normal occurrence which the individual will out-grow with time. Our study also found a significant difference in attitude along gender lines as well as participants’ level of education. We report that females were less likely than males to disagree that it was bad to wash their clothes or take a bath in the stream; this finding may be related to the gender role of females in this part of the world where women are often expected to do the laundry both for themselves and for members of their family, and in a rural setting such as the one under study, there may be no piped water at home, so it is often common place to wash and bathe in the stream. Similarly, this attitude may be influenced by the level of education, as shown by our finding, where participants with less education were more likely to support washing and bathing in the stream. Unlike the finding of Onyeneho et al.,^14^ the majority of our participants expressed a positive attitude towards seeking medical help. However, this positive attitude was not significantly associated with age, gender or level of education but rather showed significance with marital status. Our finding associated being married or cohabiting with negative attitude towards seeking orthodox medical treatment. Married and cohabiting participants preferred consulting with traditional healers. In the light of this, it was not surprising to find that married or cohabiting participants were more likely to believe that passing blood in the urine was a natural occurrence and a sign of maturity. This finding seems odd, but the reason behind it is not apparent in the study and would require further investigation.

This study found high levels of contact with water among participants. Water contact among farmworkers is usually higher than that in the general population because of the nature of farm work, which requires the regular use of water. However, the source of water is considered important because exposure to infected water could increase the risk of schistosomiasis infection. Interestingly, the main source of water used by farmworkers in this study was from the dam and the river. This high level of contact with possibly contaminated water may not necessarily imply that there were no better alternatives for the farmworkers, as it has been shown that people may have their preferences with regard to the choice of water to use on the farms.^14^ The high level of knowledge and positive attitude towards schistosomiasis prevention and control did not seem to influence the practices of the farmworkers in this study because they had different sources of water to choose from, but the majority of them chose water from the dam and the river, which are more likely to be infected. However, in corroboration with the positive attitude expressed by our participants to seek orthodox medical help in the event of schistosomiasis infection, we further reveal that indeed, the majority of those who passed blood in their urine consulted a medical doctor for treatment; this is a good practice and should be encouraged. This practice will help to control the spread of the disease and prevent complications. However, we also corroborate that in practice, married participants who passed blood in their urine were less likely to seek medical help, while both married and cohabiting participants were indeed more likely to consult a traditional healer when infected with the disease. Age and level of education were not significantly associated with the practices of participants in this study, and this shows that the level of knowledge and positive attitude may not always dictate good practices.

## Conclusion

The findings of this study show that farmworkers may be at risk for schistosomiasis in the Vuvha community. Although the level of knowledge regarding the cause of the disease was high and the majority showed positive attitude towards prevention and control of the disease, this did not influence their practices regarding schistosomiasis infection. Therefore, it is important to engage the farming communities in health education and promotion in order to control the disease.

## Limitations

This study was conducted among farmworkers in well-organised commercial farms where it is expected that optimal conditions regarding work safety would be maintained, and it is anticipated that peasant farmers’ KAP may differ. In addition, the small sample size available for analysis also poses a challenge as far as generalisation of result findings is concerned. Therefore, it is advised that this result should be interpreted with caution. Furthermore, an open-ended questionnaire would have allowed the participants the opportunity to express themselves rather than the close-ended one used in this study.

## Recommendations

1. Efforts should be made to ensure regular supply of clean water to the farms to prevent farmworkers from going to the dam and the river to fetch water. Protective equipment like rubber boots and hand gloves should be provided to avoid direct contact with contaminated water. Furthermore, through the Department of Health, the government should ensure routine testing of water samples in the communities in order to initiate purification measures were necessary.
2. Community-based studies are needed to also explore the KAP of non-farmworkers regarding schistosomiasis.
